# Biomechanical Assessment of the Collateral Ligament of the Distal Interphalangeal Joint of the Horse Following Alterations to the Palmar Angle—A Cadaveric Study

**DOI:** 10.3390/ani15030406

**Published:** 2025-02-01

**Authors:** Sandro Colla, James W. Johnson, Kirk C. McGilvray, Gustavo M. Zanotto, Kathryn A. Seabaugh

**Affiliations:** 1 Orthopedic Research Center, Department of Clinical Sciences, College of Veterinary Medicine and Biomedical Sciences, Colorado State University, Fort Collins, CO 80523, USA; katie.seabaugh@colostate.edu; 2 Orthopaedic Bioengineering Research Laboratory, Departments of Mechanical Engineering and School of Biomedical Engineering, Colorado State University, Fort Collins, CO 80523, USA; jimmy.johnson@colostate.edu (J.W.J.); kirk.mcgilvray@colostate.edu (K.C.M.); 3 Marion duPont Scott Equine Medical Center, Virginia-Maryland College of Veterinary Medicine, Virginia Tech, Leesburg, VA 20176, USA; gzanotto@vt.edu

**Keywords:** distal interphalangeal joint, collateral ligament, angle, horse, strain

## Abstract

Therapeutic shoeing for horses is used to affect the palmar angle (PA) of the distal phalanx. These changes may influence the strain on the distal interphalangeal joint collateral ligaments (DIJCLs). This cadaveric study aimed to assess DIJCL strain under varying loads and PA adjustments. Using 26 cadaver forelimbs, eight optical markers were placed on one DIJCL for strain measurement under five different loads (1000–5000 newtons) at a neutral PA, followed by PA increases of 2°, 4°, and 6° and decreases of −2° and −4°. The results indicate that increases in PA corresponded to significantly higher strain in the distal and mid distal portions, while 2° and 4° decreases in PA significantly reduced the strain in the proximal regions of the DIJCLs. Limitations include potential discrepancies between a cadaver and live biomechanics. This study concludes that increased PA increases distal DIJCL strain, while decreased PA decreases proximal strain. Further research is recommended to evaluate the practical implications of these findings in DIJCL injuries in horses.

## Simple Summary

Raising the heel of a horse is a common technique in therapeutic shoeing. This will increase the palmar angle of the distal phalanx and influence the strain on the soft tissue structures of the hoof. No previous studies have investigated the strain in different portions of the distal interphalangeal joint collateral ligaments (DIJCLs) at different palmar angles. The objective of this study was to determine the DIJCL strain under different palmar angles and loading compressions in cadaveric equine front limbs. The results show that an increase in palmar angle increases the strain on the distal portion of the DIJCLs, and a decrease in palmar angle decreases the strain in the proximal portion of the DIJCLs. Further research is needed to determine whether the information obtained in this cadaveric study can be extrapolated to live horses and used to manage the rehabilitation of horses with DIJCL injuries.

## 1. Introduction

Equine lameness is common, affecting nearly 20% of horses [1]. It represents a significant welfare concern in horses as the pain can result in early retirement, reduced mobility, and diminished quality of life. Forelimb lameness is frequently localized to the distal limb [2], which is anatomically complex and diagnostically challenging, especially when involving structures in the hoof.

Therapeutic shoeing has been the cornerstone of treatment for foot lameness in horses for decades [3,4]. Techniques have become more sophisticated, but the principles remain the same: supporting the limb of the horse in a balanced manner and relieving the strain on the injured region [3,4]. In horses with palmar heel pain, a wedged horseshoe or pad is utilized to increase the palmar angle of the distal phalanx (P3) and reduce the stress on the deep digital flexor tendon (DDFT) and navicular apparatus [3,4]. The biomechanics of this treatment have been researched in relation to the DDFT [5,6,7,8] proximal interphalangeal joint [6], superficial digital flexor tendon [6,7,8], digital joint angles [9], and kinematics of the distal forelimb [10]. This treatment, however, is not without consequence. Whilst decreasing the strain on the DDFT [5], raising the heel increases the strain on the interosseus medius muscle, clinically known as the suspensory ligament, as well as the superficial digital flexor tendon [6], and increases pressure within the distal interphalangeal joint [11].

The collateral ligaments of the distal interphalangeal joint (DIJCLs) are important stabilizers of the joint [12]. Their primary function is to support the joint in its movements in the sagittal, frontal, and transverse planes [13,14]. The prevalence of DIJCL injury is 15–30% in horses presented for magnetic resonance imaging for chronic foot lameness [15]. Injury to the DIJCLs can be diagnosed with ultrasound in 55.6% of cases [16]. This suggests that 44.4% of injuries may be undiagnosed if advanced imaging is not available. This raises the question as to whether changes to the palmar angle of P3 could influence an undiagnosed DIJCL injury.

Viitanen et al. suggest that elevated heels may be detrimental to the long-term viability of the distal interphalangeal joint (DIJ) [11]. Any alteration in the palmar angle of P3 is absorbed by adjusting the angulation of the DIJ [17], which could consequently affect the collateral ligaments of the distal interphalangeal joint (DIJCLs). The primary aim of this study was to determine the effect of different palmar angles (PAs) of P3 on the strain patterns of the DIJCLs. The first objective was to determine the strain magnitude of the proximal, middle, and distal portions of the forelimb DIJCLs with the hoof at baseline using advanced biomechanical techniques. The baseline strain magnitudes were then compared to the values obtained when the PA was increased (2°, 4°, and 6°) and decreased (−2° and −4°) from neutral. The authors hypothesized that the increased PA would increase the strain on the DIJCLs.

## 2. Materials and Methods

### 2.1. Limb Preparation

Twenty-six forelimbs (*n* = 26) from 13 horses euthanized for reasons unrelated to this study were utilized. If the hoof specimen was shod in a shoe, then the shoe was removed prior to testing. All hooves were trimmed to correct medial/lateral balance, based on lateromedial and dorsopalmar radiographs (80 KvP, mAs 0.8), using a portable digital X-ray machine (Sprint Air Equine DR, Sound Technologies, Carlsbad, CA, USA). No alteration was performed on the palmar angle prior to testing. The forelimbs were disarticulated at the metacarpophalangeal joint. The skin and deep digital flexor tendon were removed to the level of the coronary band. The superficial flexor tendon was also removed. The skin and flexor tendons had to be removed to secure the proximal phalanx into the acrylic potting system. Ligaments associated with P2 and P3 were left intact. Each limb was randomly assigned (List Randomizer, www.random.org, accessed on 12 January 2022) to have the medial (*n* = 13) or lateral (*n* = 13) DIJCL exposed. The position of the DIJCLs was determined by ultrasound. A portion of the hoof wall (2 cm × 3 cm) was removed using a motorized burr to expose the collateral ligament in its entirety. A veterinary clinician prepared all the limbs for testing. Following dissection, the limbs were wrapped in saline (0.9% NaCl)-soaked gauze to prevent the dehydration of tissues and frozen at −20 °C until undergoing testing. Twenty-four hours prior to testing, the limbs were removed from the freezer and thawed at room temperature. Biomechanical tests were performed at room temperature, and care was taken to ensure that each sample was allowed to thaw for the same amount of time. In an effort to reduce variance in the biomechanical outcomes, samples were periodically sprayed with physiological saline at 15 min intervals to ensure proper hydration. All limbs were stored in the freezer for less than 6 months [18]. The protocols utilized are well established [18] and frequently used by the coauthors (KM and JJ) [19].

The limbs from horses with known forelimb lameness localized to the foot or major radiographic abnormalities were excluded. Additionally, if the horse was shod with a wedge shoe or therapeutic shoe at the time of euthanasia, then the feet were excluded from the study. Significant abnormalities identified on radiographs or visible ligament damage during dissection also precluded the limb from being used in the study.

### 2.2. Radiography

**Figure 1:**
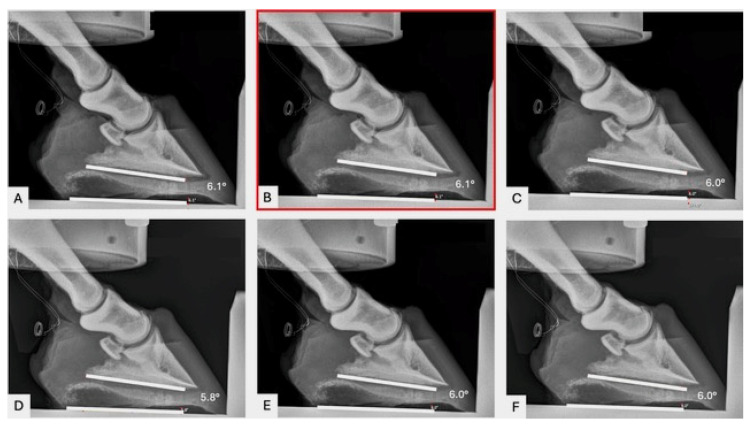
Serial radiographs of a left front foot. PA (lower right value) was measured using the radiographic imaging platform. White lines were placed on figures for visualization. (**A**) 100 newtons (N), (**B**) 1000N, (**C**) 2000N, (**D**) 3000N, (**E**) 4000N, and (**F**) 5000N. The neutral PA was measured under 1000 N of load (**B**, red box). Dorsal is to the right. Palmar is to the left. The wires visible on the left of the radiographs are the identification tags.

For all hoof specimens, lateromedial radiographs (80 KvP, mAs 0.8) were obtained with the X-ray beam centered at the hoof–ground interface [20], using a portable digital X-ray machine. This was performed after the limbs were secured in the mechanical testing system (MTS) and loaded to 100 N. Lateromedial radiographic views were repeated throughout the course of the study for every loading scenario and every wedge scenario. The PA was measured (Antech Imaging Services, Dark Horse Medical Ventures, Irvine, CA, USA) on the lateromedial radiographs by placing a line along the base of P3 and measuring the angle at which it intersected with a line parallel with the ground/platform (Figure 1).

### 2.3. Alterations to Palmar Angle

**Figure 2:**
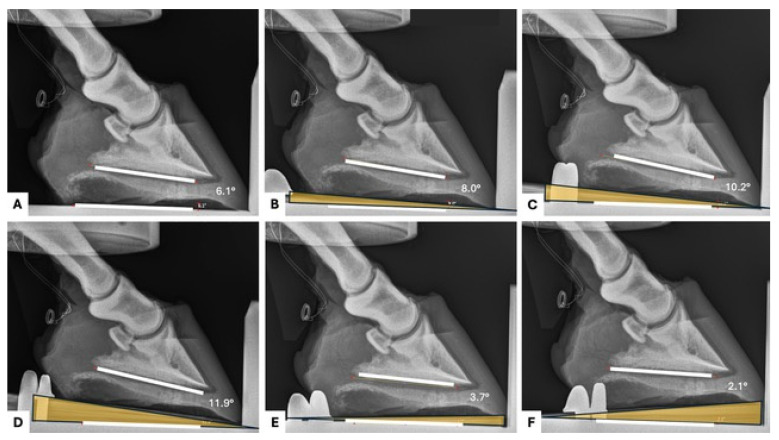
Serial radiographs of a left front foot under 1000 N of force (**A**) and with the addition of a 2° (**B**), 4° (**C**), and 6° (**D**) wedge in the heel region and a 2° (**E**) and 4° (**F**) wedge pad in the toe region of the hoof. Wedges are highlighted in yellow. PA was measured using on the radiographic imaging platform. White lines are shown on these Figures for visualization. Dorsal is to the right. Palmar is to the left.

The palmar angle that was measured at 1000 N was assumed to be that horse’s natural palmar angle (Figure 1). This angle was then altered with the addition of wedge pads (Castle Plastics, Leominster, MA, USA) placed under the sole of the hoof (Figure 2). The wedges were not secured with nails or glue. The wedges were secured to the platform with c-clamps on each side of the materials testing system. A toe plate was utilized to prevent the wedge from moving forward during loading. The addition of a 2° wedge pad represented a 2° increase in palmar angle and the same for the addition of a 4° and 6° wedge pad. To represent a reduction in palmar angle, the wedge pad was placed under the hoof backwards, with the wedge towards the toe (reversed). This elevated the toe and thereby decreased the PA. The reverse 2° wedge pad decreased the PA by 2°. A reverse 4° wedge pad decreased the PA by 4°.

### 2.4. Biomechanical Assessment

**Figure 3:**
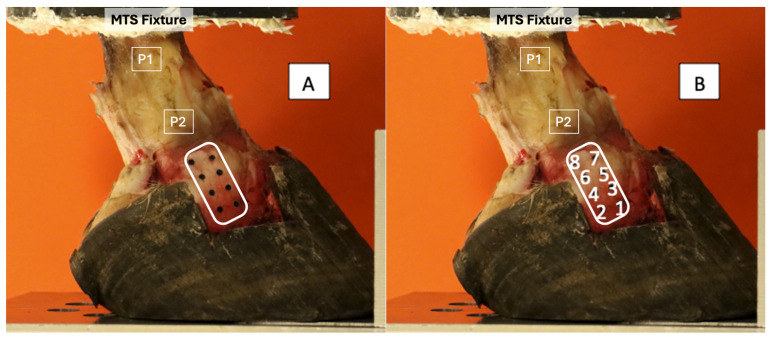
Marker placement on the exposed DIJCL (**A**) and associated numbering (**B**). The DIJCL is approximately outlined in white. Dorsal is to the right. Palmar is to the left. P1—proximal phalanx, P2—middle phalanx, MTS—material testing system.

Eight optical tracking markers (2 mm diameter electrical tape circles) were adhered to the exposed DIJCLs with super glue. Four optical markers were placed along the dorsal margin (1, 3, 5, 7) and four were placed along the palmar margin (2, 4, 6, 8) of the ligament (Figure 3) in order to measure changes in ligament length and width. Prior to the placement of the markers, the ligament was measured to ensure the symmetrical distribution of the markers over the ligament. The hoof–phalanx construct was secured to the material testing system (MTS 858 Mini Bionix, MTS Systems Corporation, Eden Prairie, MN, USA) using a custom fixture. The proximal portion of P1 was embedded in a smooth-casting resin (SmoothCast 321, Smooth On, Macungie, PA, USA) base that allowed for secure attachment to the MTS. The construct underwent simulated joint motion with compressive loads of 100 (baseline), 1000, 2000, 3000, 4000, and 5000 newtons [11,21,22,23]. Once baseline data had been acquired for an individual hoof, the same test (with all five compressive loads) was executed with a 2°, 4°, and 6° heel wedge applied to the sole of the hoof. Additionally, acquisitions were made with the 2° and 4° wedge placed in a reverse manner to elevate the toe and mimic a decreased palmar angle.

**Figure 4:**
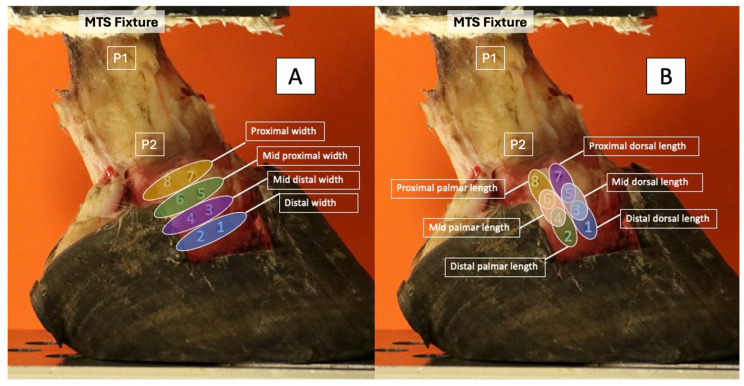
Regions of the DIJCL. Strain was measured by measuring the displacement between two selected markers and comparing to the baseline measurements (100 N) to obtain the change in width (**A**) and length (**B**). Markers 1–2, distal width; 3–4, mid distal width; 5–6, mid proximal width; 7–8, proximal width; 1–3, distal dorsal length; 2–4, distal palmar length; 3–5, mid dorsal length; 4–6, mid palmar length; 5–7, proximal dorsal length; and 6–8, proximal palmar length. Dorsal is to the right. Palmar is to the left. P1—proximal phalanx, P2—middle phalanx, MTS—material testing system.

For each testing situation, the foot underwent five loading cycles, with data collected during the 5th cycle. This loading protocol ensured that viscoelastic effects and soft tissue hysteresis were minimized. For each testing situation, photographs using a high-resolution camera (EOS 90D, Canon, Melville, NY, USA) were obtained for the subsequent measurement (NIH Image J program, U.S. National Institutes of Health) of the marker displacement between two neighboring markers. The camera was secured by a tripod and kept in the same place throughout the data collection. The distances between the markers were measured, and the strain was calculated by the change from the baseline (100 N) associated with each region of the ligament (Figure 4). The equation for strain was as follows: ε = ΔL/L_0_. The change in length (ΔL) = L_0_ − L_x_. L_0_ was the length between markers under 100 N of load. L_x_ was the length between the same markers for each of the testing scenarios (neutral, +2°, +4°, +6°, −2° and −4°. “Baseline” strain was calculated from changes in marker distances from 100 N to 1000 N. A strain value that was greater than baseline strain for the same ligament region was considered an increase in strain; a value that was less than baseline was considered a decrease in strain.

### 2.5. Statistical Analysis

The data were assessed for normality using a Shapiro–Wilk test. JMP software (JMP^®^, Version 17, SAS Institute Inc., Cary, NC, USA) was used for statistical analysis. A mixed model was fit separately for each pair of tracking markers. “Wedge” (reverse 4, reverse 2, 0, 2, 4, 6° wedge), “newtons” (1000, 2000, 3000, 4000, 5000), and “wedge × newton” interaction were included as fixed effects. “Left or right” was included as a random effect to account for repeated measures on each leg. Terms that showed evidence of difference based on F-tests underwent Dunnett adjusted pairwise comparisons for wedges and Tukey adjusted pairwise comparisons for different forces (N). Microsoft Excel (Version 16.85) was used to organize the data. Significance was defined at *p* < 0.05.

## 3. Results

The average neutral palmar angle, calculated on radiographs with the limbs loaded to 1000 newtons to mimic normal loading during the stance phase, was 5.14° (*n* = 26, range: 0.7° to 8.7°; SD = 2.44). The lowest PA was −3.3° after the addition of a 4° toe wedge. The greatest PA was 14.7° following the addition of a 6° heel wedge. Due to a neutral palmar angle lower than 4°, 5 of the 26 feet developed a negative palmar angle after the addition of 4° toe elevation. In total, 3 of the 26 feet developed a negative palmar angle after the addition of 2° toe elevation.

**Figure 5:**
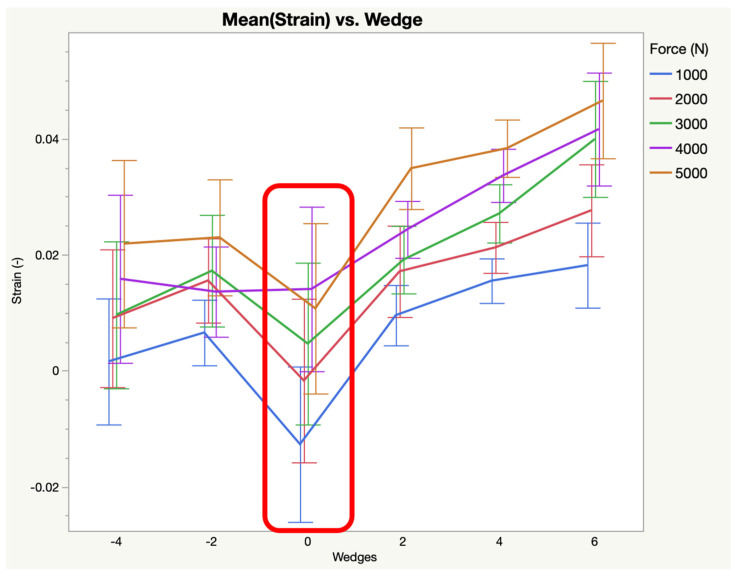
Mean strain versus wedges for the distal width of the DIJCL (markers 1–2). Baseline strain (neutral PA) is highlighted with the red square. All values greater than baseline are increases in strain. All values less than baseline are decreases in strain.

**Figure 6:**
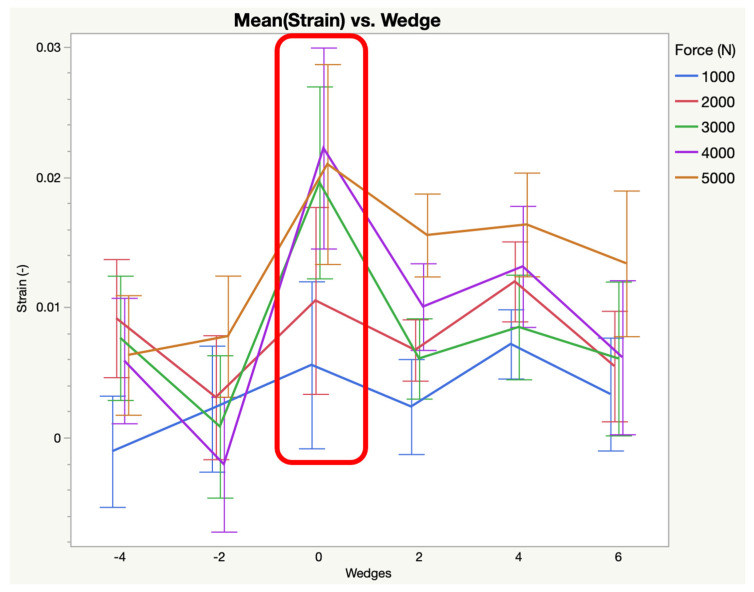
Mean strain versus wedges for the distal palmar length (markers 2–4). Baseline strain (neutral PA) is highlighted with the red square. All values greater than baseline are increases in strain. All values less than baseline are decreases in strain.

The 6° increase in palmar angle resulted in increased strain in the distal width (0.0350 (±0.0061), *p* < 0.0001) and mid distal width (0.0404 (±0.0072), *p* = 0.0003) and decreased strain in the distal palmar length (0.0069 (±0.0033), *p* = 0.0007). The 4° increase in palmar angle resulted in increased strain in the distal width (0.0271 (±0.0061), *p* < 0.0001) and mid distal width (0.0348 (±0.0072), *p* = 0.0373). The 2° increase in palmar angle resulted in increased strain in the distal width (0.0210 (±0.0061), *p* < 0.0001) and decreased strain in the distal palmar length (0.0082 (±0.0033), *p* = 0.0035). A 2° decrease in palmar angle resulted in increased strain in the distal width (0.0168 (±0.0062), *p* = 0.0145) and decreased strain in the distal palmar length (0.0027 (±0.0033), *p* < 0.0001), proximal dorsal length (−0.0377 (±0.0038), *p* = 0.0336), proximal palmar length (−0.0688 (±0.0071), *p* = 0.0005) and proximal width (0.0019 (±0.0058), *p* = 0.0029). A 4° decrease in palmar angle resulted in decreased strain in the distal palmar length (0.0059 (±0.0033), *p* = 0.0002), mid dorsal length (−0.0042 (±0.0056), *p* = 0.0003), proximal dorsal length (−0.0404 (±0.0038), *p* = 0.0008), proximal palmar length (−0.0738 (±0.0071), *p* < 0.0001), and proximal width (0.0020 (±0.0058), *p* < 0.0001). Table 1 provides the resulting mean strain (±standard error) for each region of the ligament across all loads following the addition of wedges. The changes to the mean strain of the distal width of the DIJCL in response to wedges is represented in Figure 5. The changes to the mean strain in the distal palmar length of the DIJCL in response to wedges is represented in Figure 6.

## 4. Discussion

In the current study, the authors hypothesized that increasing the palmar angle (PA) of the distal phalanx would increase strain in the DIJCLs. The results confirmed this hypothesis. Increasing the palmar angle resulted in increased strain in the distal portion of the ligament, as demonstrated by significantly increased strain in the distal width, distal palmar length, and mid distal width. Conversely, however, decreasing the palmar angle also influenced the strain in the DIJCLs, resulting in decreased strain in the proximal portion of the ligament, as demonstrated by significantly decreased strain in the proximal palmar length, proximal dorsal length, and proximal width. Both of these findings could be clinically applicable.

The DIJCLs in horses originate from the distal medial and lateral aspects of the middle phalanx and insert on the dorsomedial and dorsolateral surfaces of the distal phalanx [24]. They are critical stabilizer structures that support the distal interphalangeal (DIJ) joint during movements in multiple planes [12,13,14]. The distal interphalangeal joint is a highly complex area. The DIJCL is a key ligament, but there are other ligaments that are closely associated with the DIJCL [14,25]. The chondrocoronal ligament originates on the dorsal aspect of the middle phalanx in close proximity to the origin of the DIJCLs. Additionally, there are periligamentous fibers of the DIJCLs that attach to the ungual cartilages. Lastly, the DIJCL is closely associated with the joint capsule of the DIJ [14]. The authors did not evaluate the influence of the PA of the distal phalanx on these other structures. The authors felt that the DIJCL was a more identifiable ligamentous structure and therefore more clinically applicable.

Strain represents the relative change in the ligament shape or size in response to an applied force. In the current study, changes in ligament shape were quantified by displacement between eight optical tracking markers in response to the applied force, as well as changes in PA. Ligament strain is also influenced by the magnitude, duration, and angle of applied forces. Clinically, uneven ground or hoof conformation can influence strain patterns by altering the magnitude, duration, and angle of applied forces, potentially leading to the overloading of specific ligament fibers. Chronic overloading or an acute overload incident can result in damage to collagen fibers, reducing the ligament’s elasticity and strength and increasing the risk of injuries such as desmopathy or tears.

The results of the current study should be considered when making recommendations for a horse with a known DIJCL injury. A reduction in strain in an injured DIJCL would be beneficial in promoting adequate healing, especially during the initial phases of rehabilitation. Based on the current study, reducing the PA would be beneficial in a horse with desmitis of the proximal portion of the DIJCL. Throughout the recovery process, the angles can be managed for a gradual increase in strain in the affected DIJCL, aiming to return to normal PA values. It is important to note that advanced diagnostic imaging such as magnetic resonance imaging (MRI) or computed tomography is required to better characterize the location and extension of DIJCL injuries. The incidence of DIJCL injuries identified with MRI ranges from 12 to 75% of cases with lameness localized to the foot [26,27,28]. The DIJCL is most commonly injured in the distal portion of the ligament. The identification of DIJCL injuries without advanced imaging is difficult since the distal ½–¾ of the ligament is located within the hoof capsule, making visualization with ultrasonography impossible [16]. Desmopathy of the distal portion of the DIJCL is often underdiagnosed due to the inability to diagnose without advanced imaging. Therefore, the addition of a wedge for therapeutic shoeing could increase strain in an undiagnosed DIJCL injury.

The average palmar angle in the forelimb has been reported as 2–10° [29]. Dyson et al. reported the average PA in 300 horses as 5.63° [30]. The wedges selected for the current study were chosen based on clinical experience and would be reasonable therapeutic shoeing considerations. Since toe elevations are less commonly prescribed, only 2° and 4° wedges were used. The previous literature shows that an increase in PA is beneficial in injuries related to the navicular region and the deep digital flexor tendon (DDFT); however, based on the current study, an increased PA also increases the strain of the distal portion of the DIJCL, which is a potential disadvantage in lesion healing. A decrease in the PA of P3 decreases the strain in the superficial digital flexion tendon and interosseus medius muscle apparatus [5,6,7,8,9,10]. Based on the current study, a decrease in PA could reduce the strain on the proximal portion of the DIJCL. It is important to consider that multiple injuries may exist within the same hoof. The results of the current study, as well as prior studies [16,27,28,31], show that changes to the palmar angle with trimming or wedge pad placement should be pursued with caution and with a thorough knowledge of the anatomy of the foot.

The peak vertical force for a 450 kg horse is approximately 1800 newtons (N) when standing, 3600 N when walking, and 5400 N when trotting during the stance phase of each limb [23]. It was based on these values that we selected the forces for the current project. The application of 5000 N is representative of 0.84 body weight for a 450 kg horse at a trot rather than the referenced 0.9 [23]. While we appreciate that this loading range did not cover the entire physiological loading regime, it is important to note that this load was kept consistent across groups, allowing a direct comparison among treatments. Herein, we are not reporting the expected physiological strain on the ligament but rather the difference in strain as a function of 5000 N. Additionally, we did not collect weights for all the horses prior to euthanasia. We therefore based our target forces on what has been reported. The authors felt that increasing the force to 6000 N would have exceeded the strength of the construct and would have resulted in deformation beyond what would have occurred physiologically. The results of the current study could be extrapolated to a horse undergoing walk and trot. Due to the cadaveric nature of the study, however, we cannot take into account the dynamic aspect of the stride, including the large “slide” of the hoof–ground interaction at higher speeds and the influence of different ground surfaces. The linear increases in force did result in linear alterations in strain, suggesting that the effects on the DIJCL would be more pronounced as speed increases.

The limitations of this project are associated with the inherent differences between the real-life biomechanics and ex vivo biomechanics of cadaver limbs. The disarticulation of the limb through the metacarpophalangeal joint and the removal of soft tissue structures, especially the DDFT, possibly altered the biomechanics of the distal interphalangeal joint, as the DDFT applies palmar support to the joint. The goal in disarticulating at the metacarpophalangeal joint was to remove the influence of other joints, so that the differences in strain patterns between trials would only be induced by the change in the palmar angle. The DDFT was lacking normal function and integrity without its proximal muscle attachment, and therefore, it was removed. An additional limitation was that the rigid attachment of the hoof to the platform did not mimic the initial slide portion of the stride that is present in vivo. The majority of horses also land first with the lateral heel, whereas in this study, the sole was secured in a flat position, and forces were applied symmetrically in the proximal-to-distal sagittal plane. The transection of a portion of the hoof wall to expose the DIJCL might affect the hoof biomechanics as well; this should also be considered when discussing the translatability to live horses. Future work should include finite element analysis to further explore the effect of the palmar angle on strain in this complex structure.

Despite these limitations, the goal of this project was to provide a reference regarding the strain patterns in DIJCLs with the proposed model to provide proof of concept. These preliminary data provide the groundwork to further explore the role of hoof conformation, as well as different therapeutic shoes, on this important structure in the equine locomotive apparatus.

## 5. Conclusions

In conclusion, in this ex vivo cadaveric study, it was shown that an increased palmar angle may be detrimental to the distal portion of the DIJCL, and a negative palmar angle may actually be beneficial to injuries to the proximal portion of the DIJCL due to the decreased strain in this region. Due to the cadaveric model, the data should be applied with caution to real-life scenarios. The data obtained from this study provide essential information for further research focused on corroborating those findings with results obtained from live horses and potentially using them to guide corrective shoeing in horses diagnosed with DIJCL injuries during their rehabilitation process.

## Figures and Tables

**Table 1:** Mean strain (standard error) as measured by displacement for each region of the DIJCL following changes in the palmar angle. Strain was calculated as ε = ΔL/L_0_. Change in length (ΔL) = L_0_ − L_x_. L_0_ was the length between markers under 100 N of load. L_x_ was the length between the same markers for each of the testing scenarios (neutral, +2°, +4°, +6°, −2° and −4°).

	Changes to Palmar Angle					
Region of the Ligament (Associated Marker Numbers)	6° Wedge	4° Wedge	2° Wedge	Neutral	Reverse 2° Wedge	Reverse 4° Wedge
Distal width (1–2)	0.0350 * (±0.0061)	0.0271 * (±0.0061)	0.0210 * (±0.0061)	0.0036 (±0.0061)	0.0168 * (±0.0062)	0.0114 (±0.0061)
Distal dorsal length (1–3)	0.0182 (±0.0044)	0.0204 (±0.0043)	0.0163 (±0.0044)	0.0207 (±0.0044)	0.0237 (±0.0044)	0.0240 (±0.0044)
Distal palmar length (2–4)	0.0069 * (±0.0033)	0.0114 (±0.0033)	0.0082 * (±0.0033)	0.0159 (±0.0033)	0.0027 * (±0.0033)	0.0059 * (±0.0033)
Mid distal width (3–4)	0.0404 * (±0.0072)	0.0348 * (±0.0072)	0.0300 (±0.0072)	0.0274 (±0.0073)	0.0307 (±0.0073)	0.0341 (±0.0073)
Mid dorsal length (3–5)	0.0022 (±0.0056)	0.0052 (±0.0055)	0.0062 (±0.0056)	0.0087 (±0.0056)	0.0020 (±0.0056)	−0.0042 * (±0.0056)
Mid palmar length (4–6)	−0.0379 (±0.0085)	−0.0366 (±0.0085)	−0.0399 (±0.0085)	−0.0389 (±0.0085)	−0.0424 (±0.0085)	−0.04015 (±0.0085)
Mid proximal width (5–6)	0.0152 (±0.0061)	0.0106 (±0.0060)	0.0072 (±0.0061)	0.0138 (±0.0061)	0.0105 (±0.0061)	0.0148 (±0.0061)
Proximal dorsal length (5–7)	−0.0282 (±0.0037)	−0.0259 (±0.0037)	−0.0265 (±0.0037)	−0.0288 (±0.0038)	−0.0377 * (±0.0038)	−0.0404 * (±0.0038)
Proximal palmar length (6–8)	−0.0527 (±0.0071)	−0.0549 (±0.0071)	−0.0609 (±0.0071)	−0.0564 (±0.0071)	−0.0688 * (±0.0071)	−0.0738 * (±0.0071)
Proximal width (7–8)	0.0114 (±0.0058)	0.0110 (±0.0058)	0.0099 (±0.0058)	0.0127 (±0.0058)	0.0019 * (±0.0058)	0.0020 * (±0.0058)

* Strain that was statistically different (*p* < 0.05) compared to the strain calculated at the neutral PA.

## Data Availability

Raw data were updated at Dryad and are available at https://datadryad.org/stash/share/q53dOWJuwls8QW6uuFQTA8IljoV0Hlm3LAZiBpgGQeA (accessed on 28 January 2025).
